# Is the eye-movement field confused about fixations and saccades? A survey among 124 researchers

**DOI:** 10.1098/rsos.180502

**Published:** 2018-08-29

**Authors:** Roy S. Hessels, Diederick C. Niehorster, Marcus Nyström, Richard Andersson, Ignace T. C. Hooge

**Affiliations:** 1 Experimental Psychology, Helmholtz Institute, Utrecht University, Utrecht, The Netherlands; 2 Developmental Psychology, Utrecht University, Utrecht, The Netherlands; 3 Lund University Humanities Lab, Lund University, Lund, Sweden; 4 Department of Psychology, Lund University, Lund, Sweden; 5 Tobii AB, Stockholm, Sweden

**Keywords:** eye movements, fixation, saccade, eye tracking, definitions

## Abstract

Eye movements have been extensively studied in a wide range of research fields. While new methods such as mobile eye tracking and eye tracking in virtual/augmented realities are emerging quickly, the eye-movement terminology has scarcely been revised. We assert that this may cause confusion about two of the main concepts: fixations and saccades. In this study, we assessed the definitions of fixations and saccades held in the eye-movement field, by surveying 124 eye-movement researchers. These eye-movement researchers held a variety of definitions of fixations and saccades, of which the breadth seems even wider than what is reported in the literature. Moreover, these definitions did not seem to be related to researcher background or experience. We urge researchers to make their definitions more explicit by specifying all the relevant components of the eye movement under investigation: (i) the oculomotor component: e.g. whether the eye moves slow or fast; (ii) the functional component: what purposes does the eye movement (or lack thereof) serve; (iii) the coordinate system used: relative to what does the eye move; (iv) the computational definition: how is the event represented in the eye-tracker signal. This should enable eye-movement researchers from different fields to have a discussion without misunderstandings.

## 1. Introduction

Movements of the eyes have been measured since the early 1900s [1,2], and have since been studied widely. As such, eye movements have long been used to draw inferences about perception, cognition and brain function in many areas of psychology, cognitive science and applied research fields (e.g. [3,4]). Two events (or concepts) that are prominent in a large part of the eye-movement literature are *fixations* and *saccades*. They are *events* in the sense that they are periods (with a start, end and a duration) into which the eye-tracker signal is often divided—on occasion supplemented by other events such as blinks or smooth pursuit. These events have been discussed for a long time. Indeed, as Wade [5] points out, the term *saccade* had already been adopted into the English literature back in 1916 by Raymond Dodge. This means that this term has been used for at least 100 years. Assuming that our knowledge of eye movements has increased since 1916, one might also assume that the terminology^1^ has been updated to reflect the scientific advances in the eye-movement research field. Or perhaps the meaning of the terminology has remained unchanged for over a century? We find the latter hard to believe. Alternatively, we hypothesize that the usage of the terms *fixation* and *saccade* is currently shrouded in conceptual confusion. This seems to be a recurring problem, and, for example, in psychology it has previously occurred around the concepts of ecological validity [6] and the stimulus [7]. Although Gibson [8] writes about the various and inconsistent usage of the term stimulus, his words apply here as well: ‘It is convenient and easy to do so, but if the words are slippery and if we allow ourselves to slide from one meaning to another unawares, we are confused without knowing it’ (p. 49). We wonder whether we are confused without knowing it in eye-movement research as well. In this paper, we investigate to what degree eye-movement researchers agree in their definitions of fixations and saccades. To be clear, our goal is not to review studies on one or more properties of fixations or saccades, as others have done extensively before [9–11], but rather to assess how the concepts of ‘fixations’ and ‘saccades’ are defined and used in the field of eye-movement research, and to highlight opportunities for improvement.

We hypothesize that fixations and saccades are shrouded in conceptual confusion based on several (interrelated) arguments. First, we consider several typical scenarios of eye-movement research to illustrate how fixations and saccades may broadly be defined. These examples should serve to show that different definitions may be used across different fields and within one field. Hereafter, we will argue that confusion may stem from at least three possible sources: (i) different frames of reference, (ii) functional versus oculomotor versus computational definitions of fixations and saccades, and (iii) event classification in the eye-tracker signal. Finally, we consider whether these sources of confusion may be minimized, and introduce the survey we conducted among 124 eye-movement researchers. Although we will focus extensively on the concepts of fixations and saccades, many of the issues we address are likely to hold for concepts such as *dwell*, *glance*, *gaze* and others that are used in the literature (see [9, p. 190] for a discussion).

### 1.1. Typical eye-movement research scenarios

#### 1.1.1. Scenario 1: eye movements during reading

Reading research is a popular field of eye-movement research, and has been so for long [4]. In this line of research, fixations may correspond to the period of uptake of visual information (in this case, text), and saccades are movements of the eyes that bring other parts of the text onto the most sensitive part of the retina for information uptake. Typically, high-speed eye trackers are used (greater than 250 Hz) that offer high accuracy (low systematic errors) so that the point of regard can accurately be mapped to sentences, words or even characters. One example of an eye tracker that is often used is the SR Research EyeLink 2 (e.g. [12,13]), of which the output is a gaze coordinate on the screen.^2^ In reading research, head movements are typically minimized by the use of a chin and/or forehead rest. In order to label parts of the eye-tracker signal thus recorded as fixations and saccades, computational definitions are constructed (generally implemented using an algorithm). A fixation may, for example, be considered as a cluster of gaze coordinates within a specified range in space and time, whereas a brief peak in the velocity signal of the gaze signal may correspond to a saccade. Note that the thresholds for what constitutes a computational fixation or saccade may be determined by the dimensions of the text that was presented. As Rayner [4] states: ‘For example, most researchers lump together successive fixations that are on adjacent characters as a single fixation’ (p. 374).

#### 1.1.2. Scenario 2: eye movements of infants

Eye tracking is also popular among developmental psychologists, for whom infants' eye movements may be considered ‘a window on cognitive development’ [14], particularly when the infants are pre-verbal. As infants cannot easily be restrained in their movement (as compared with the use of a chin or forehead rest in adult research), remote eye trackers are typically employed so that the infant is relatively free to move around. A typical example is the Tobii TX300 remote eye tracker (e.g. [15,16]), which also outputs a gaze coordinate on the screen. While fixations are generally considered as a proxy for cognitive processing in infant research as well, there are several caveats. Infants cannot be instructed to perform a certain task, and are prone to staring. Therefore, it is typically questionable to what degree ‘cognitive processing’ takes place. Moreover, infants' eye-tracking data are prone to a larger variable error (lower precision), for example, due to infant movement [17]. This consequently requires computational definitions of fixations that are specifically designed to deal with large variable error in the eye-tracker signal [18].

#### 1.1.3. Scenario 3: eye movements during ambulatory vision

The scenarios sketched above were limited to screen-based eye tracking with sedentary participants, which does not afford locomotion. Two classic examples of eye-tracking studies in which participants could freely move their head and move around are reported by Land *et al.* [19] and Hayhoe [20]. In these studies, participants were fitted with a head-mounted mobile eye tracker that recorded both a video of the eye and a video of the visual scene from the perspective of the participant during an everyday task. Here, unlike in the previous scenarios, both the vestibulo-ocular reflex (VOR) and the optokinetic reflex—both compensatory eye movements based on vestibular and visual information, respectively, in order to counteract movements of the head, self or large-scale movements in the visual world—will be represented in the eye-tracker signal, as the eye tracker measures eye rotation relative to the head. In other words, the head-mounted eye tracker reports gaze coordinates in a head-centred frame of reference (e.g. a gaze coordinate in the scene video), whereas the eye trackers in the two previous scenarios report gaze coordinates in a world-centred frame of reference (e.g. a gaze coordinate on the screen). Applying the same computational definitions of fixations and saccades to this scenario as in the previous scenarios may not be sufficient. For example, a brief peak in the velocity signal which serves as a computational definition in scenario 1, may also be recorded as a compensation to a sudden head movement (VOR) in this scenario. We will consider the different frames of reference in more depth as the first potential source of confusion.

### 1.2. Source of confusion 1: frames of reference

Remote or tower-mounted eye trackers generally output a gaze coordinate with reference to a computer screen, for example pixel *x* horizontally and pixel *y* vertically on screen (scenarios 1 and 2). These coordinates are world-centred (the screen being a fixed position in the world). Mobile eye trackers, however, generally output a gaze coordinate with reference to the scene video, for example, pixel *x* horizontally and pixel *y* vertically in the video frame (scenario 3). In this case, the coordinates are head-centred; they are with reference to the scene video which moves around as the participant moves her head. The frames of reference of these two types of eye trackers are different—world-centred versus head-centred. Such frames of reference may also be termed eye-in-head and gaze-in-world/eye-in-space (e.g. [21–23]). Different frames of reference pose a problem for defining fixations and saccades [24].

Consider a participant that sits still and is asked to fixate a small object depicted in the centre of the computer screen in front of her. Should her eye movements be recorded with a remote eye tracker, her gaze coordinate will be in the centre of the screen. As she starts to move her head from left to right, her gaze coordinate should remain in the centre of the screen. Although her eyes may rotate with respect to her head (head-centred), the world-centred point of regard remains the same, which is what the eye tracker reports. Now consider the situation when the participant is wearing a head-mounted mobile eye tracker. As she sits still and gazes at the centre of the screen, the gaze coordinate will be (roughly) in the centre of the scene video (assuming this corresponds to the centre of the screen). Should she now move her head while continuing to fixate the centre of the screen, the gaze coordinate will change with the movement, as the eye tracker reports gaze coordinates with respect to the head-fixed scene video. In both cases, the task for the participant is to fixate, but each case is represented quite differently in the eye-tracker signal. As stated, the same computational definitions of a fixation will not suffice for eye-tracker signals from different frames of reference: a velocity peak in the gaze coordinate signal from a mobile eye tracker need not reflect the same movement of the eye as a velocity peak in the gaze coordinate signal of a remote eye tracker. However, in some software packages from eye-tracker manufacturers, it is possible to apply the exact same algorithm for classifying fixations and saccades to data from remote and mobile eye trackers, which can lead to eye-movement metrics that seem comparable (as they involve the same terminology), but are actually incomparable (as the underlying signals are fundamentally different). Granted, the ambiguity in a velocity profile of a head-centred gaze measurement with regard to whether the eye or the head moves can be resolved by adding other sources of information. For example, one might use an accelerometer or video record to determine whether the head or eyes move with respect to the environment. Such information sources are generally not featured, however, in algorithms that allow automatic eye-tracking data analysis (but see [25,26]).

That different frames of reference can lead to implicit conceptual confusion is evident from the literature. In some cases, the term ‘fixation’ is used both in world-centred and head-centred frames of reference, even in the same research field, without explicitly distinguishing between the two. In infant looking behaviour, for example, Merin *et al.* [27] report on fixations recorded with a remote eye tracker, whereas Franchak *et al.* [28] report on fixations recorded with a mobile eye tracker. This is problematic for two reasons. First, when properties of fixations from both studies are discussed without considering the coordinate system, a nonsensical comparison is made. For example, when comparing fixation durations between infants of different ages and different methods, confusion may arise. Infants aged a few months are less capable of disengaging attention and shifting gaze to a new fixation location than older infants, such that their ‘fixations’ are generally of longer duration. ‘Fixations’ may, however, also be of shorter or longer duration if the same algorithm is applied to eye-tracking data from a remote or mobile eye tracker. Second, the oculomotor system is heavily under development just after birth, such that infants develop the ability to pursue moving objects with respect to the environment within the first year [29]. Particularly, here it is vital to be specific on what constitutes a ‘fixation’, and whether the ‘fixation’ target is moving with respect to the infant or not, in order to avoid potential confusion due to concepts being underspecified. Next we examine whether there is less ambiguity if events are defined from a functional point of view.

### 1.3. Source of confusion 2: functional, oculomotor or computational definitions

Table 1 contains examples of definitions of fixations and saccades as specified in the literature. This is not an exhaustive overview, but merely a collection of definitions to illustrate potential problems in how fixations and saccades are defined. Note that some of the works cited are highly influential in eye-movement research.^3^ Interestingly, researchers define fixations in various ways, for example, at a functional level (e.g. [18,38]), where Gegenfurtner *et al.* [39] add how its function is achieved (i.e. by miniature eye movements). Others, define a fixation as a period during which the eye is relatively still [9,40]; i.e. an oculomotor characteristic. Saccades are also defined in various ways, for example at a functional level (e.g. [2,11,18,33,35,38]), or by describing (oculomotor) characteristics of saccades [4,37]. There are several ambiguities to be found in this overview:

(1) As stated, fixations may be defined at a functional level or as the eye being relatively still (relative to the head, although this is not explicit in the definitions). This brings situations to mind in which the definitions of fixations are contradictory. For example, when a participant fixates an object and moves around in the room, this might be considered a fixation under the former functional definitions of a fixation, yet not under the latter ‘eye-stillness’ definitions, as the eye rotates with respect to the head. The frames of reference as outlined above seem to be critical here. Fixation as a ‘stillness’ of the eye relative to the head only occurs when the fixation target remains stationary with respect to the head. If there is any movement of the head relative to the world while the same point in the world is fixated, the orientation of the eyes will change (thereby not being still with respect to the head). The functional definition of stabilizing a target relative to the fovea can apply to both targets being stationary or moving with respect to the head.
(2) Saccades are described on several occasions as the inter-fixation interval [9,40]. This not only means that the definition of a saccade depends on the definition of a fixation, but it also seems to imply one of two research scenarios. If anything else than a saccade is considered a fixation, it may be that this definition captures, for example, smooth pursuit and VOR as well. This is reasonable, if one considers all forms of image stabilization relative to the retina as fixation. An alternative scenario would be when one considers the interval between saccades a fixation, but implicitly holds that smooth pursuit and VOR are not a fixation. In this case, the scenario is likely that of an eye tracker providing world-centred gaze coordinates (here, VOR is not picked up) using only stationary fixation targets (smooth pursuit does not occur). Given the fact that Holmqvist *et al.* [9] and Larsson *et al.* [40] hold an ‘eye-stillness’ definition of fixation, the latter scenario likely applies to them, whereas the former may apply to, for example, Hessels *et al.* [18] or Jovancevic-Misic & Hayhoe [41].

The highly influential work of Leigh & Zee [11] may be particularly relevant in the context of functional definitions and frames of reference. They give the following functional description that ‘… eye movements are of two main types: those that stabilize gaze and so keep images steady on the retina and those that shift gaze and so redirect the line of sight to a new object of interest’ (p. 5). Whereas some researchers seem to consider the first type of eye movements as they are defined functionally as equivalent to a fixation, others only consider holding the image of a *stationary* object (in world-centred coordinates) on the fovea as a fixation. See Leigh & Zee [11], who state that visual fixation ‘Holds the image of a stationary object on the fovea by minimizing ocular drifts’ (p. 5).

**Table 1.** Example descriptions of fixations and saccades. Examples are chosen that describe or define fixations and saccades without referring to previous work, or combine multiple studies into one description. Bold-face is used to highlight fixation or saccade.

authors	description
Dodge [2]	describes five types of eye movements (of which type 1 described a **saccade** [5]) ‘… whose sole function is to move the line of regard to an eccentric point of interest’ (p. 316)
Bahill *et al.* [30]	‘**Saccades** are the fast, staccato eye movements characteristically displayed by people who are reading or looking about a scene’ (p. 107)
Inchingolo & Spanio [31]	‘fast position correcting eye movements, called **saccades** or quick phases of nystagmus. The former refers to voluntary refixation movements …’ (p. 683)
Rayner [4]	‘**Saccades** are rapid movements of the eyes with velocities as high as 500° per second.’ ‘Between the saccades, our eyes remain relatively still during **fixations** for about 200–300 ms’ (p. 373)
Salvucci & Goldberg [32]	‘… **fixations** (pauses over informative regions of interest) and **saccades** (rapid movements between fixations)’ (p. 71)
Sparks [33]	‘… generates high-velocity movements (**saccades**) of both eyes that bring the image of the target onto or near the fovea’ (p. 953)
Ramat *et al.* [34]	‘**Saccades** are the rapid eye movements used to voluntarily move gaze from one target of interest to another’ (p. 11)
Duchowski [35]	‘**Fixations** are eye movements that stabilize the retina over a stationary object of interest’ (p. 46). ‘**Saccades** are rapid eye movements used in repositioning the fovea to a new location in the visual environment’ (p. 42)
Falkmer *et al.* [36]	‘**Saccades** are ballistic movements, 20–150 ms long, reaching a velocity up to 800° s^−1^. Saccades […] direct the eye so that external visual objects are projected onto the fovea. Here they are processed with high precision by means of **fixation** […]. Fixation time corresponds to cognitive processing time and can vary from 80 ms […] to about 500 ms’ (p. 711)
Shic *et al.* [37]	‘… **saccades** (rapid, ballistic movements of the eye) and **fixations** (periods where the point of regard by the eye is spatially relatively stable)’ (p. 1)
Rolfs [38]	‘**Saccades** (rapid eye movements), on the one hand, aim for visual information currently outside the fovea. **Fixations**, on the other hand, keep a target relatively stable with respect to the photoreceptors on the retina’ (p. 2415)
Holmqvist *et al.* [9]	‘ … the state when the eye remains still over a period of time […]. This is called a **fixation** …’ (p. 21). ‘The rapid motion of the eye from one fixation to another […] is called a **saccade**’ (p. 23)
Gegenfurtner *et al.* [39]	**Fixation** is defined as ‘Miniature eye movements that relatively stabilize the retina for a prolonged posture of the eyes over an object’ (p. 526)
Kowler [10]	‘**Saccadic eye movements** are the rapid shifts of the line of sight made to bring the fovea—the centre of best vision—from one selected location to another’ (p. 1466)
Leigh & Zee [11]	‘Visual **fixation**—Hold the image of a stationary object on the fovea by minimizing ocular drifts. **Saccades**—Bring images of objects of interest onto the fovea’ (p. 2)
Larsson *et al.* [40]	‘**Fixations** are periods when the eye is more or less still, while **saccades** are fast movements between the fixations that take the eyes from one object of interest to the next’ (p. 145)
Hessels *et al.* [18]	‘There is a primary distinction between the periods in which an area of the visual scene is kept on the fovea—a **fixation**—and periods in which an area of the visual scene is brought onto the fovea—a rapid eye position change called a **saccade**’ (p. 1803)

To sum up this brief overview, fixations and saccades are defined in the literature at different levels, and there seem to be contradictions between definitions that can be related to the function of eye movements and the frames of reference in which they are recorded. If the sole problem is that fixations or saccades may be defined at different levels, it may be easily remedied by explicating the categories as *functional*, *oculomotor* and *computational* fixations or saccades. Of course it may be (as our scenarios should show) that different researchers may use different definitions of fixations and saccades at both functional, oculomotor and computational levels. As Hooge *et al.* [42, p. 13] state on oculomotor, functional and computational definitions:

There is not one simple definition for a fixation; some definitions are formulated as a combination of properties (duration, frequency, amount of small movements), some are functional (e.g. to help perception) or are formulated as a recipe to detect fixations. It is to be expected that human coders have different internal representations, ideas about or definitions of fixations.

Indeed it seems to be the case that researchers have different internal representations, as Inhoff & Radach [43] write based on an informal survey of 32 eye-movement researchers that ‘… two thirds of them consider the definition of functional oculomotor events controversial …’ (p. 30). As the previous scenarios should also have clarified, a researcher may use concurrent definitions of fixations and saccades at a computational level and a functional (or oculomotor) level. Using both a computational and a functional definition of a fixation is, of course, a reasonable thing to do. A computational definition may be used to classify parts of the eye-tracker signal as ‘fixation’, which a functional definition does not do. However, confusion may arise if definitions are used interchangeably or are not made explicit (i.e. when it is unclear *what* fixation is referred to). This is a genuine problem, as the different definitions do not correspond to the *same* thing. As Holmqvist *et al.* [9, p. 150] state on the issue:

In reality, perfect matches between the fixations detected by an algorithm and moments of stillness of the eye [i.e. the definition of a fixation as used in this book] are very rare. To make matters worse, the term fixation is sometimes also used for the period during which the fixated entity is cognitively processed by the participant. The oculomotor, the algorithmically detected, and the cognitive ‘fixations’ largely overlap, but are not the same.

Holmqvist *et al.* [9] make clear that functional, oculomotor and computational fixations do not necessarily correspond to the same event, neither temporally nor conceptually. However, there are clear examples of how the different definitions of fixation are related to each other. For example, applying a computational definition to the eye-tracker signal can serve to estimate some oculomotor change (e.g. a rotation of the eye). Classifying fixations or saccades by using features of the eye-tracker signal is usually referred to as event detection, which may be a potential source of confusion by itself.

### 1.4. Source of confusion 3: event classification in the eye-tracker signal

In the eye-movement literature, so-called event-detection algorithms [9] are often described that are meant to detect fixations or saccades in the eye-tracker signal. First of all, the term event *detection* is misleading, and may have contributed to the confusion. Event detection presumes that an oculomotor event (be it fixation or saccade) is objectively present in the eye-tracker signal and all one needs to do is detect where it is. The eye-tracker signal, however, does not consist of a succession of oculomotor events, but rather of a succession of features (e.g. position of the pupil or corneal reflection) measured from the eye using an eye tracker that may be indicative of these events. This distinction is important. For example, if one were to take ‘saccades’ as ‘detected’ by an algorithm to be true saccades, one might draw wrong conclusions, e.g. that saccades in the dark are slower than saccades in the light. If, on the other hand, one considers the feature which is measured from the eye by the eye tracker, in this case the pupil, it turns out that the apparent slowing of saccades in the dark is an artefact of the eye-tracking technique based on said feature [44]. Eye movements may appear quite differently in the eye-tracker signal, depending on the type of eye tracker used to measure them [45]. An algorithm, therefore, does not so much detect, rather than computationally define events such as fixations and saccades (cf. [46]) by virtue of the computations in the algorithm. Henceforth, the term event *classification* is to be preferred. A computationally defined fixation is not the same as a conceptual fixation (be it functionally defined or otherwise).

Although any event-classification algorithm inherently provides a computational definition of a fixation or saccade (as it is an algorithm designed to label part of the eye-tracker signal as such), it is interesting to note that the authors of such recent algorithms do not explain their *a priori* model for what constitutes a fixation or a saccade (e.g. [47–49]). In recent machine-learning approaches (e.g. [50]), the problem may be larger as the computational definition may not be fully explicit: e.g. if it is based on eye-tracking data coded by human coders, of whom we are not sure what rules were applied [42]. In any case, there are many different event-classification algorithms available, for example, to classify fixations and saccades in the eye-tracker signal from different types of eye trackers (or participant groups), as already alluded to above. However, it is unclear whether the authors of different event-classification algorithms hold different definitions at the conceptual level as well, and whether these definitions affect the computational definitions of fixations and saccades. In other words, do the computational fixations from the different event-classification algorithms correspond to the same conceptual fixation?

In order to illustrate the problem further, the difference between the computational and conceptual levels may be put bluntly: a computational definition is essentially meaningless with regard to the function of a fixation or saccade. It is meaningless in the sense that a computational definition only results in numbers, e.g. number of fixations and the corresponding durations. But what these values entail depends on the conceptual definition of *what* a fixation reflects, for example, an index of some cognitive process in a specific task. Unless a conceptual definition is available in order to provide this meaning, the numbers themselves remain meaningless. Of course these values (e.g. an average fixation duration) can be meaningful to researchers, but this is because the researchers bring with them (implicit) background knowledge and context. Without a researcher's context or a conceptual definition, a number is nothing more than a number.

Finally, as the temporal and spatial resolution of eye trackers increases, it becomes possible to see much more detailed characteristics of the eye-tracker signal. One such characteristic is what has been termed the post-saccadic oscillation (PSO) [51–53]. Recent work suggests that PSOs blur the line between fixations and saccades [45,48,52], which poses additional problems for defining fixations and saccades at the computational level.

### 1.5. The conceptual confusion considered

We have discussed three potential sources of confusion that indicate eye-movement research, in general, seems to be suffering from a muddled terminology: frames of reference, different levels of definition and event classification. The terms *fixations* and *saccades* may refer to functional, oculomotor and computational events, which may differ between the research fields in which the terms are applied. Definitions in the literature are sometimes contradictory, and can be traced back to certain frames of reference or research settings. Confusion may arise particularly when different definitions are used interchangeably. One might assert, then, that the ‘conceptual confusion’ is particularly a problem of specification. As Andersson *et al.* [54] rightly point out, ‘… despite fuzzy definitions, researchers talk about fixations, saccades, and other events at conferences with apparent ease. So there must be some intuitions between experts on what events occur in a given stream of data …’ (p. 620). How can it be that there is such an apparent mismatch between the ease of discussion at conferences and the status quo in the literature? Is it the case that the explicit definitions in the research literature are ill-stated, but clear and consistent definitions are implicitly used in the field?

To investigate this, we surveyed 124 eye-movement researchers about their definitions of fixations and saccades. Our first question was whether researchers are as divided on the definitions of fixations and saccades as the literature seems to suggest, and whether researchers' definitions can be predicted by their background or expertise. If it can, we might conclude that clear definitions are used in different sub-fields of eye-movement research, or by more experienced researchers. If this division cannot be traced back to, for example, the research field in which they are active, then this attests to the necessity of the explication of fixation and saccade definitions. Our second question was whether researchers are divided on certain well-defined event-classification problems, both theoretically, and at the level of the eye-tracker signal. It has been suggested that PSOs, for example, blur the line between fixations and saccades (e.g. [45]), or that noise affects how fixations are classified in the eye-tracker signal (e.g. [18]). If researchers are divided at the level of the eye-tracker signal, this attests to the necessity of making computational definitions of fixations and saccades explicit. Our third question was whether there are preferred definitions of fixations and saccades, and whether the choice for a specific definition can be predicted by researchers' experience. In answering these questions, we will refer back to the three sources of confusion we have identified: frames of reference, different levels of definition and event classification.

## 2. Methods

### 2.1. Participants

Participants in our survey were recruited via two routes. First, eye-movement researchers that we know and considered to be knowledgeable in the field were invited to participate. Second, all attendees of the 19th European Conference on Eye Movements (ECEM) held in the summer of 2017, at the Bergische Universität Wuppertal, Germany, were invited to participate in our survey. All corresponding authors as identified in the conference proceedings were contacted and asked to participate (*n* = 239), and to pass on the link to the survey to colleagues they considered to be knowledgeable. A total of 124 usable responses to the survey were recorded, of which 112 were complete.

#### 2.1.1. Eye-movement researchers' background and experience

In order to determine whether our sample of eye-movement researchers was diverse and represented at least a good part of the eye-movement research community in general, we assessed (1) how long they have been active in eye-movement research, (2) which sub-field of eye-movement research they most identified with and (3) what types of eye trackers and software they worked with. At the time of the survey, participants had been active in eye-movement research on average 9.66 years (median = 8.50 years, s.d. = 7.09 years). Table 2 depicts the sub-fields of eye-movement research that participants most identified with. The categories were based on the thematic sessions in the programme of the 18th ECEM held at the Universität Wien, Austria, in 2015. What should be evident from this overview is that there is a distribution across different fields and that no single field exceeds 15% of the sample. Of the 112 participants that completed the full survey, 63% use tower-mounted eye trackers, 74% use remote eye trackers, 38% use mobile eye trackers and less than 10% use EOG, scleral coils or other eye-tracking techniques. Moreover, 62% of the participants analyse eye-tracking data using eye-tracker manufacturer software, 25% using software described in the literature and 58% using self-written software. Note that multiple answers were possible, so that these values need not sum to 100%.

**Table 2.** Number of participants who identified with each sub-field of eye-movement research.

field	no. responses
attention	17
perception	13
reading	12
scene perception	12
eye-tracking methodology	11
educational science	7
expertise	3
neuropsychology	3
usability	3
development	2
clinical groups	2
social influences	2
neurophysiology	2
visual search	2
language	1
microsaccades	0
other	20

Based on the diverse background of the eye-movement researchers that participated in the present survey, we conclude that we have a diverse sample of the eye-movement research community, at least the community represented at the bi-annual ECEM. It might be, however, that we have an overrepresentation of researchers who analyse eye-tracking data using self-written software. In any case, such an overrepresentation might actually benefit the responses collected, as these researchers have implemented computational definitions of their own.

### 2.2. Survey

The survey was conducted online using the open source software LimeSurvey, version 2.05+, build 141229. It consisted of four parts, progressing from open-ended questions with no additional information provided to fully closed questions with all the relevant information provided. After completing each part, participants could not revisit their answers to previous questions.

The first part was meant to assess what researchers consider to be fixations and saccades. It consisted of two questions: ‘what is a fixation?’ and ‘what is a saccade?’. Researchers were free to make their answers as elaborate as they wished. The answers were coded by the authors as follows:

(1) Authors R.H. and I.H. went through all the answers and noted all characteristics of fixations/saccades that were mentioned by the participants, what functions these fixations/saccades fulfil, and what restrictions apply to the definition.
(2) The coding scheme resulting from the previous step was sent to all authors, each of whom used the scheme to annotate one-fifth of the answers.
(3) Problem cases were discussed, missing categories were added, and a new coding scheme was drafted.
(4) All authors re-coded the answers they had previously coded with the updated scheme.
(5) Each author checked the answers coded by one of the other coders.
(6) Disagreements were first discussed between each pair of coders, and resolved when coders could come to agreement.
(7) All remaining disagreements were resolved in a general meeting.

**Figure 1.**
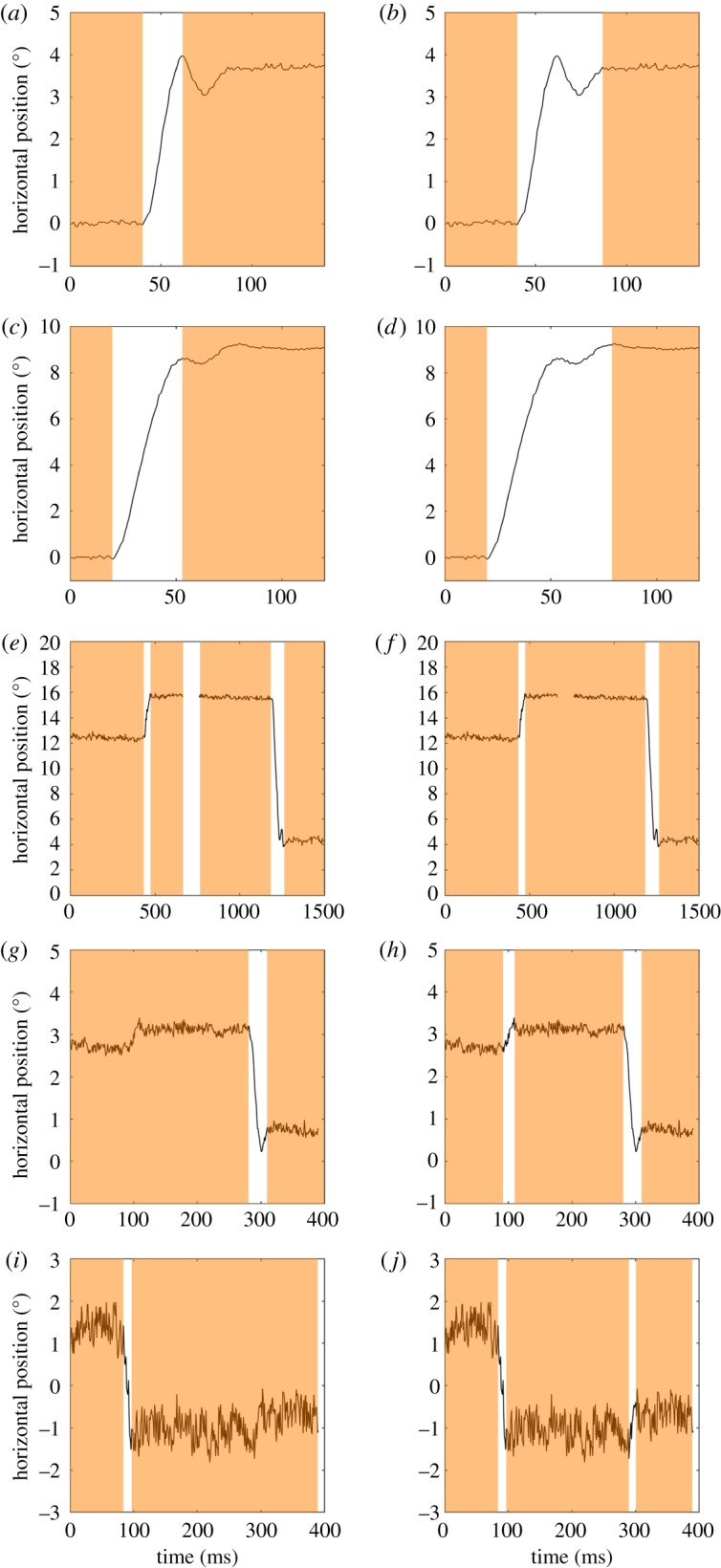
Examples of fixations marked with orange bars in eye-tracking data, as presented to participants with the question: Which of the following two examples most accurately reflects fixations in the eye-tracking data? Each row represents one example, with each column representing a possible set of fixations. The examples contained a post-saccadic oscillation (*a*,*b*), undershoot (*c*,*d*), data loss (*e*,*f*), a small saccade in low-noise data (*g*,*h*) and high-noise data (*i*,*j*).

The second part of the survey was meant to assess how divided researchers were on certain well-defined problems, both at the level of the eye-tracker signal, as well as theoretically. This part of the survey consisted of five examples of eye-tracking data (figure 1), each with two alternatives on how fixations were labelled in the data. The data examples address the third potential source of confusion: event classification. Participants had to indicate which of the two alternatives best reflected the fixations in the data. Additionally, two yes/no questions were posed that address the first potential source of confusion: frames of reference. The problems were as follows:

(1) Is a PSO coded as part of the fixation or not (figure 1*a*,*b*)?
(2) Is an undershooting saccade with a PSO coded as part of the fixation or not (figure 1*c*,*d*)? Examples 1 and 2 were included, as previous literature has suggested PSOs blur the line between fixations and saccades [45,48,52].
(3) Does a small loss of eye-tracking data break up a fixation (figure 1*e*,*f*)? Data loss is a problem prevalent in, for example, developmental eye-tracking [17]. Whether data loss is seen to break up fixations or not is an important question for fixation-classification purposes [18].
(4) Does a small saccade break up a fixation (figure 1*g*,*h*)? Previous research has shown that eye-tracking researchers may set different thresholds as to what constitutes a fixation-breaking eye movement [42].
(5) Does a small saccade embedded in noise break up a fixation (figure 1*i*,*j*)? The same eye-tracking data from the previous example were taken, inverted in direction to prevent recognition, with noise added to the data. Does the same displacement still count as a fixation-breaking eye movement, if the noise level is higher? This is an important question for fields where eye-tracking data may be noisier (e.g. developmental eye tracking).
(6) ‘If one continuously looks at an object while one walks around, do you consider this “fixation”?’. This question is meant to assess whether a ‘eye-stillness’ definition of fixation (e.g. [9]) is widely held by the participants or not. If an observer moves while looking at the same object, there is presumably no stillness of the eyes, as the eyes rotate to keep the object fixated during movement (barring the exception where both observer and object move simultaneously in the same direction).
(7) ‘If one continuously looks at an object while the object moves, do you consider this “fixation”?’. This question is meant to assess whether observer and object movement are considered as two separate classes of movement. If researchers answer no to this question, but yes to the previous question, this means that what entity moves (object versus observers) matters for the definition of a fixation.

The third part of the survey was meant to assess whether certain definitions of fixations and saccades are predominantly used in eye-movement research. For both fixations and saccades, several alternatives based on the eye-movement literature were posed (cf. table 1), and participants were asked to indicate which definition they agreed with the most.

The fourth and final part consisted of several background questions (as described in the Participants section), and allowed participants to enter any comments.

## 3. Results

### 3.1. What are fixations and saccades according to eye-movement researchers?

In order to assess how researchers define fixations and saccades from the top of their minds, we coded their answers to the two open questions ‘what is a fixation?’ and ‘what is a saccade?’. The full coding scheme is given in table 3 and depicts the number of times each code was assigned to an answer. Codes are grouped by overarching categories. As visible from table 3, physical characteristics of fixations and saccades were often noted by participants, whereas references to the function or coordinate system were much less common. While this overview represents how participants described fixation and saccades overall, the question arose to what degree certain combinations of categories were consistently coded. Is it more likely, for example, that one who noted the coordinate system was also more likely to describe the function of a fixation or saccade? In order to investigate this, we computed *r*_*ϕ*_, a measure for the association between binary variables (corresponding to Pearson's correlations estimated for binary variables). *r*_*ϕ*_ was calculated for each combination of coded categories, separately for fixations and saccades.

**Table 3.** Coding scheme for annotating open answers with number of codes in bold.

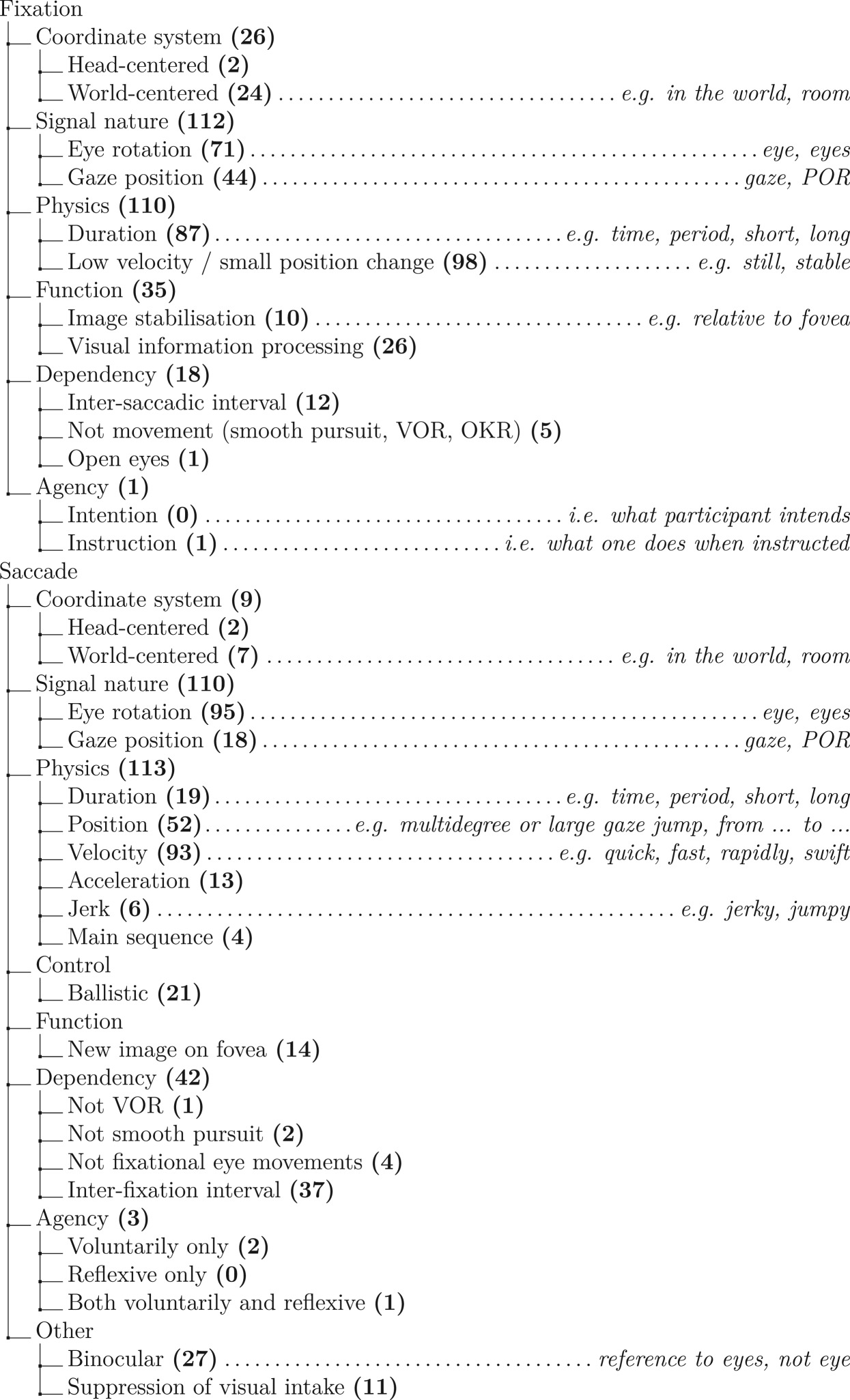

**Figure 2.**
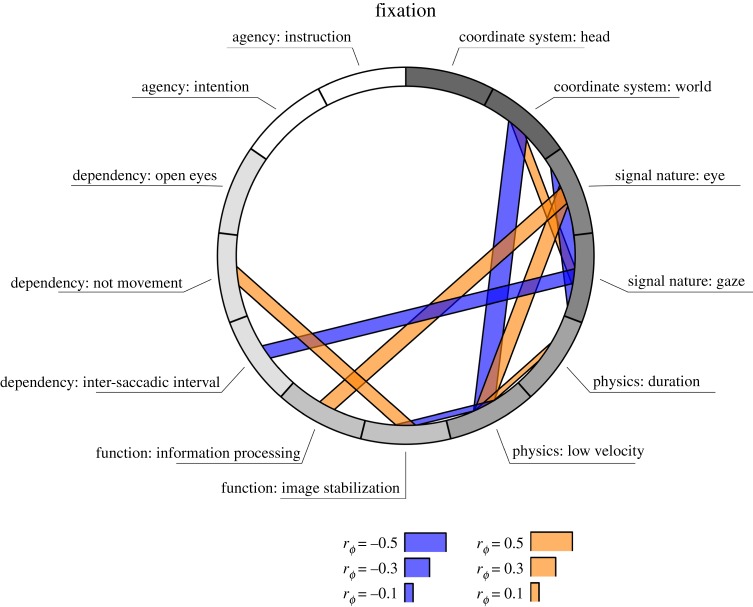
Associations between coded categories for the responses to the question what is a fixation? For visualization purposes, only those associations which yielded significant coefficients at *p* < 0.05 are depicted. Negative associations are depicted by blue bars, positive associations by orange bars. The width of the bar indicates the strength of the association, with the full width of one circular element corresponding to a *r*_*ϕ*_ of 1 or −1.

**Figure 3.**
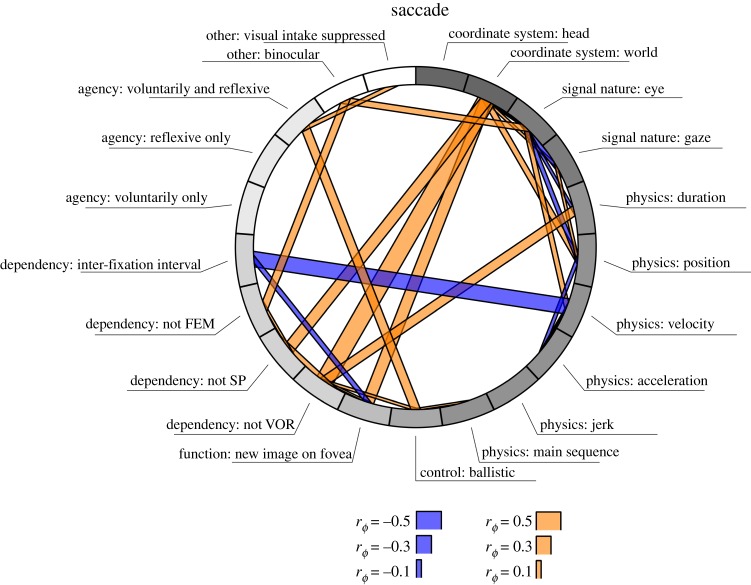
Associations between coded categories for the responses to the question what is a saccade? For visualization purposes, only those associations which yielded significant coefficients at *p* < 0.05 are depicted. Negative associations are depicted by blue bars, positive associations by orange bars. The width of the bar indicates the strength of the association, with the full width of one circular element corresponding to a *r*_*ϕ*_ of 1 or −1.

Figures 2 and 3 depict the associations between coded categories for fixation and saccades, respectively. As we were interested in determining gross patterns in our survey data, we have visualized only *ϕ*-coefficients that are significant at an alpha level of 0.05. This significance level is only a way of selecting the prevailing patterns without being conservative, not to test any specific hypotheses. Specific hypotheses about the survey data are tested using Bayesian methods later on. As visible from figure 2, participants who referred to the eye (as opposed to gaze) as the nature of the signal were also more likely to note something about the low velocity profile, and the information-processing function of fixations. Participants who gave their response in a world-centred coordinate system were, on the other hand, less likely to note a low velocity profile during fixation. As visible from figure 3, the strongest associations were observed for the world-centred coordinate system. If participants noted their response in a world-centred coordinate system, they were also more likely to specify the function of a saccade (directing the fovea to a new part of the visual scene), and the fact that saccades need to be distinguished from smooth pursuit and VOR. Participants who noted the saccade as being the interval between fixations were less likely to note anything about the velocity of a saccade. Although there are some associations between the different categories, it does not appear to be the case that there is a clear systematic relationship between whether participants noted function, coordinate system, or physical properties of fixations and saccades.

A second question that arose was whether participants' answers were related to their background: which eye trackers or analysis software researchers use, or in which field they are active. Is it the case, for example, that participants who are active in neurophysiological research are more likely to note physical properties, whereas participants who are active in attention research, are more likely to note functional properties?

**Figure 4.**
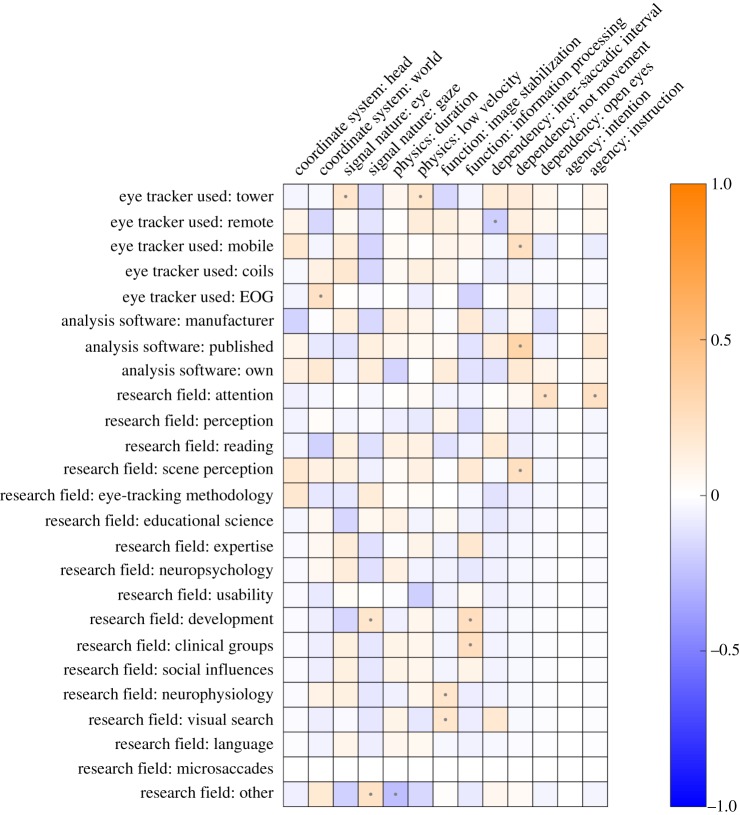
Associations between coded categories for the responses to the question ‘what is a fixation?’ and researcher background (eye tracker used, analysis software used and research field identified with). Questions on researcher background are treatment-coded; e.g. all participants who identified most with the research field reading are coded as 1 for that variable, while the rest are coded as 0. Negative associations are depicted in blue, positive associations in orange. The stronger the association, the brighter the colour. Associations which yielded significant coefficients at *p* < 0.05 are depicted with a small dot.

**Figure 5.**
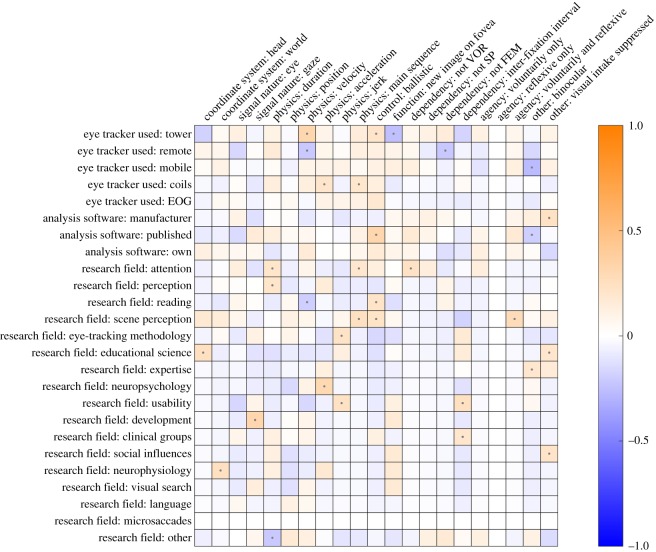
Associations between coded categories for the responses to the question ‘what is a saccade?’ and researcher background (eye tracker used, analysis software used, and research field identified with). Questions on researcher background are treatment-coded; e.g. all participants who identified most with the research field reading are coded as 1 for that variable, while the rest are coded as 0. Negative associations are depicted in blue, positive associations in orange. The stronger the association, the brighter the colour. Associations which yielded significant coefficients at *p* < 0.05 are depicted with a small dot.

**Figure 6.**
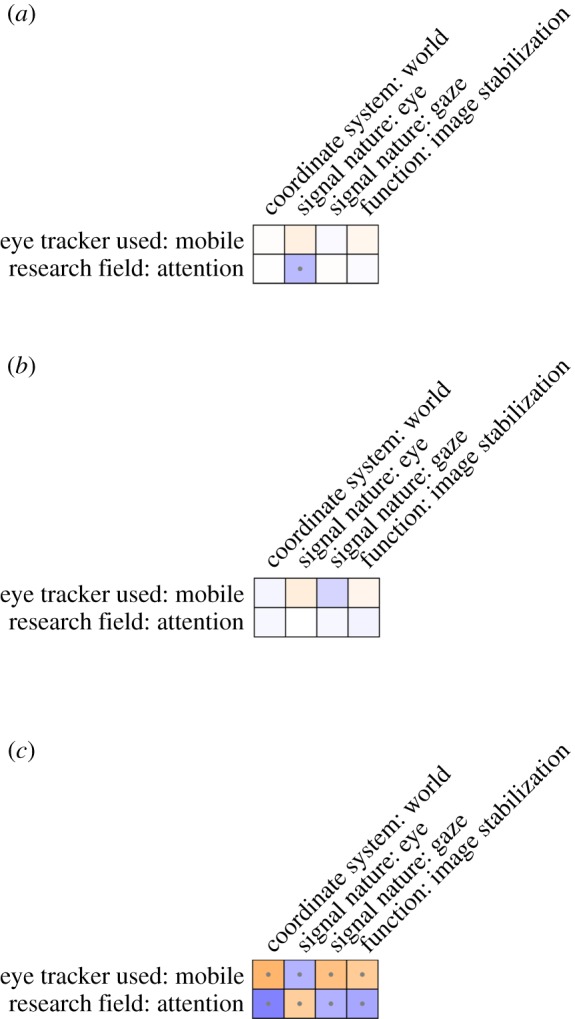
Associations between four example coded categories for the responses to the question ‘what is a fixation?’ and two example variables on researcher background. Associations are given for three scenarios: the data from the survey from which the coded categories were shuffled (*a*), the real data (*b*) and an ideal scenario (*c*). Negative associations are depicted in blue, positive associations in orange. The stronger the association, the brighter the colour. Associations which yielded significant coefficients at *p* < 0.05 are depicted with a small dot.

Figures 4 and 5 depict the associations between coded categories for fixation and saccades, respectively, and researcher background. As an example, consider participants who identified as using tower-mounted eye trackers. These participants (1) were more likely to note the eye as the signal nature for fixations, (2) were more likely to note the low velocity profile for fixation, (3) were more likely to note the velocity of saccades, (4) were more likely to describe the ballistic characteristic of saccades, and (5) less likely to note the function of saccades, compared with participants not using tower-mounted eye trackers. Note, however, that the coefficients are quite low overall. In order to provide some context for these coefficients, two benchmarks are depicted in figure 6. The top panel depicts a version of the real data for which the responses were shuffled. This should reflect the range of coefficients we may expect based on noise alone. The bottom panel depicts an idealized scenario. This idealized scenario is based on hypothetical associations. For example, the association between users of mobile eye trackers and reporting a world-centred coordinate system is computed under the assumption that 80% of the mobile eye-tracking users note this, while only 20% of the non-mobile eye-tracking users note this. The real data seem to be much more comparable to the shuffled data than to the ideal data. This seems to suggest that there are no clear relationships between researcher background and coded response data.

While researcher background did not seem to predict which categories were coded, it may be that more experienced participants were more likely to provide a nuanced and elaborate answer. To investigate whether this is the case, we conducted a Bayesian correlation in JASP [55] between the number of years that researchers were active in the field to the number of categories coded from their answers. The hypothesized positive correlation was not supported by the data (BF_+0_ = 0.52). In order to investigate whether more experienced researchers were more likely to have specific categories assigned to their response, the set of 112 completed questionnaires was divided into two groups according to the median number of years active in eye-movement research (Group 1: > 8.5 years and Group 2: ≤ 8.5 years). Two-by-two Bayesian contingency tables were analysed in JASP for all coded categories with the hypothesis that Group 1 ≠ Group 2. There was no category for which this hypothesis was supported. All Bayes factors indicated evidence in favour of the null model. In almost all cases, this was with a BF_01_ > 3, with the highest BF_01_ = 14.51. For the following four categories, the BF_01_ was between 1 and 3: (1) information processing for a fixation, (2) low velocity for a fixation, (3) image stabilization for a fixation and (4) not fixational eye movements for a saccade. For these categories, there was barely any evidence in favour of the null hypothesis, though the null hypothesis was better supported than the hypothesis that the two groups were unequal.

In sum, there are many different aspects of fixations and saccades that were noted by participants. There were not one or two definitions that prevailed. Definitions did not seem to be related to researcher background or experience.

### 3.2. Well-defined problems for fixation and saccade definitions

In response to the five eye-tracking data examples (figure 1) and questions, participants answered as follows:

(1) The presence of PSOs for classifying fixations: 21% of the participants assigned the PSO to the fixation (panel *a* as opposed to panel *b* in figure 1), whereas 40% of the participants assigned the undershooting saccade with PSO to the fixation (panel *c* as opposed to panel *d* in figure 1).
(2) Classifying fixations in the presence of data loss: 71% of the participants allowed data loss, and assigned it to a fixation (panel *f* as opposed to panel *e* in figure 1).
(3) The effect of noise on classifying a fixation: 69% of the participants let a small saccade break up a fixation in low-noise data (panel *h* as opposed to panel *g* in figure 1). Only 29% of the participants let a small saccade break up a fixation in high-noise data (panel *j* as opposed to panel *i* in figure 1).
(4) Observer and object movement for the classification of fixations: 64% of the participants consider it to be ‘fixation’ when an object is continuously looked at, while the observer moves. Only 32% of the participants consider it to be ‘fixation’ when an object is continuously looked at, while the object moves.

There seem to be favourites for at least four of the eye-tracking data examples (not counting the example containing undershoot with a PSO). Moreover, participants are more inclined to consider observer movement as integral to fixation, whereas object movement is less likely to be considered so. However, there are substantial disagreements for some of the examples or questions.

### 3.3. Which fixation and saccade definitions were chosen most often?

**Figure 7.**
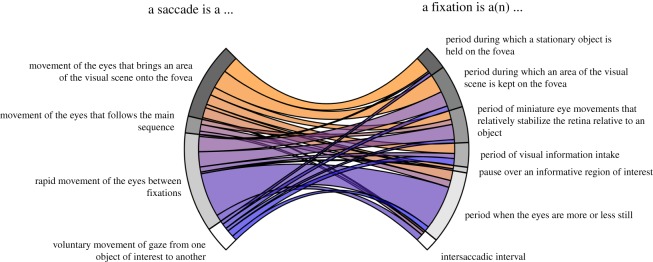
Definition of fixations and saccades that participants were asked to decide between. The circular elements at either end of the diagram reflect the proportion of participants picking that specific definition. Connecting bands reflect the proportion of participants that picked the corresponding definition at the other end of the diagram.

Participants decided between four saccade and seven fixation definitions with which they agreed most. Figure 7 depicts the proportions of participants that picked each definition, as well as how choices for fixation and saccade definitions were related to each other. Among saccade definitions, the functional definition and the inter-fixation interval were selected most often. Among fixation definitions, the ‘eye-stillness’ definition was selected most often, although there was quite some spread among the alternatives. Interestingly, participants who chose the inter-fixation interval definition of saccades were most likely to pick the ‘eye-stillness’ definition of fixations. Participants who picked the functional definition of saccades were more likely to pick functional definitions of fixations. More so than with the open-ended question in part 1 of the survey, two classes of definitions seem to arise here.

In order to determine whether researchers' experience predicted which definition was chosen, a 2 (experience group) by 7 (fixation definition) Bayesian contingency table was analysed in JASP, as before with the hypothesis that Group 1 ≠ Group 2. No definite evidence for or against this hypothesis was observed (BF_01_ = 1.63). Similarly, a 2 (experience group) by 4 (saccade definition) Bayesian contingency table was analysed, again with the hypothesis that Group 1 ≠ Group 2. Here, the null model was supported as opposed to the hypothesis that the two groups were unequal (BF_01_ = 11.60), indicating that researcher experience was not related to which saccade definition was considered best.

Finally, participants were asked whether their idea about fixations and saccades had changed while filling in this survey, to which 26% of the participants answered ‘yes’.

## 4. Discussion

There seems to be a mismatch between the ease with which researchers discuss fixations and saccades at conferences (as surmised by Andersson *et al*. [54]) and the state in the literature of how these events are defined. To investigate whether researchers are as divided in the definitions of fixations and saccades as the literature seems to suggest, we surveyed the definitions held by 124 eye-movement researchers. Specifically, we investigated (1) the breadth of fixation and saccade definitions held by eye-movement researchers and whether these can be predicted by researcher background or experience, (2) whether researchers are divided on well-defined theoretical problems and problems at the level of interpretation of the eye-tracker signal and (3) whether there are preferred definitions of fixations and saccades, and whether the choice for a specific definition can be predicted by researchers' experience. In answering these questions, we will refer back to the three potential sources of confusion that we have identified in the Introduction: frames of reference, different levels of definition (functional, oculomotor, computational) and event classification.

We have shown that eye-movement researchers hold a variety of definitions of fixations and saccades, of which the breadth seems even wider than what is reported in the literature. When open-ended questions were asked, eye-movement researchers generally specified physical (oculomotor) characteristics of fixations and saccades, whereas functional descriptions (source of confusion 2) or references to the coordinate system (source of confusion 1) in which the eye movement occurs were scarce. Researcher background or experience was not systematically related to what definitions of fixations or saccades were reported. This means that we did not observe any evidence suggesting that clear definitions are held in sub-fields of eye-movement research. When researchers were asked to pick the definition of a fixation and saccade that they agreed with the most, eye-movement researchers were divided primarily between functional definitions, and a ‘eye-stillness’ definition of a fixation combined with an ‘inter-fixation interval’ definition of a saccade. Which definition was picked was not related to researcher experience. The two alternatives may, however, be related to implicit frames of reference used (source of confusion 1), to which we will return shortly.

When presented with well-defined problems at the level of the eye-tracker signal, eye-movement researchers were divided on how fixations should be marked when PSOs, undershoot, data loss or a high level of noise are present. There seemed to be clear favourites for some examples (e.g. 79% of the eye-movement researchers responded that a PSO should not be coded as a fixation^4^). This underlines the fact that definitions of fixations and saccades need to be stated explicitly, both at the computational level as well as at the conceptual level. We cannot assume that everyone agrees whether a PSO should be classified as distinct from a saccade or fixation, or (hierarchically) as part of a fixation or saccade, nor can we assume that everyone agrees on what a saccade or fixation is. As such, clarifying the definitions should reduce the potential confusion due to using different levels of definition interchangeably (source of confusion 2) or assuming that eye-movement events are objectively present in the eye-tracker signal (source of confusion 3). An example of the latter is the fact that eye ball rotation during saccades is not accurately captured by pupil-CR eye trackers (e.g. [52]).

When asked whether observer movement or object movement is allowed under the definition of a ‘fixation’, there appeared to be controversy. Two-thirds of the eye-movement researchers allowed observer movement to be present under the definition of a fixation, whereas one-third did not. Only one-third of the eye-movement researchers allowed object movement to be present during a fixation, whereas two-thirds did not. The controversy here may be related to the problem we introduced in the Introduction. Does one define fixation as continuously looking at a stationary object with respect to the world, or does one define fixation as the stabilization of an area of the visual scene with respect to the retina (regardless whether observer and/or the object move)?

In sum, our survey shows the eye-movement field may indeed be suffering from confusion in the form of a muddled conceptual framework, and attests to the necessity for clarifying some of the conceptual confusion in the eye-movement terminology. We will first discuss the main problems for eye-movement research as uncovered in our study, whereafter we suggest how the conceptual confusion may be minimized. One of the main problems observed is that fixations and saccades can be defined at various levels (e.g. functional, oculomotor or computational), which can cause confusion if these definitions are used interchangeably. This might be easily mitigated by having future research be explicit about the level at which fixations or saccades are defined. However, it appears that the problem runs deeper, particularly with regard to the definition of fixations. We have identified at least two complicating factors, being (1) implicit frames of reference and (2) fixations as stabilization of gaze on any object versus fixations as the stabilization of gaze on *stationary* objects with respect to the world only. In order to clarify this, we revisit some fixation definitions from the literature.

### 4.1. Fixation definitions revisited

We have, on several occasions, referred to an ‘eye-stillness’ definition of a fixation, which is epitomized by Rayner [4] who states that ‘Between the saccades, our eyes remain relatively *still* during fixations’ (p. 373) and Holmqvist *et al.* [9], who state that: ‘… the state when the eye remains *still* over a period of time […] is called a fixation …’ (p. 21). The fact that ‘stillness’ is problematic, is already noted by Rayner [4, p. 373] himself, who states:

Although researchers interested in eye movements in information processing tasks typically discuss fixations as the period of time when the eyes are still, the term fixation is something of a misnomer. The eyes are never really still, because there is a constant tremor of the eyes …

However, we believe that the term fixation is not the misnomer, but that ‘still’ is problematic here. ‘Still’ only has meaning, if one specifies ‘still’ in relation to something. Specifically, is it the case that the eye does not rotate relative to the head, or relative to an object fixated? It may be that these definitions are implicitly linked to the research setting: eye-tracking research using tower-mounted eye trackers or remote eye trackers with participants in a chin rest. Here, fixating a stationary object on the screen means that the orientation of the eyes relative to the head and object does not change (or very little if we consider fixational eye movements; in other words ‘relatively still’). This emphasizes that it is necessary for researchers to specify the coordinate system that they are using, in order to avoid conceptual confusion. Does one mean ‘stillness’ of the eye in head-centred, object-centred or in world-centred coordinates?

A second problem identified in the Introduction (and confirmed in our survey) is the following. Leigh & Zee [11] have described that: ‘… eye movements are of two main types: those that stabilize gaze and so keep images steady on the retina and those that shift gaze and so redirect the line of sight to a new object of interest’ (p. 5). As stated, some researchers seem to consider all (or most) of the eye movements that ‘stabilize gaze and so keep images steady on the retina’ as a fixation. Others, including Leigh & Zee [11], only consider holding the image of a *stationary* object with respect to the world on the fovea as a fixation. The important difference seems to be whether an object moves relative to the observer, or whether an observer moves relative to an object. Duchowski [35, p. 46] states on the issue:

Fixations are eye movements that stabilize the retina over a stationary object of interest. It seems intuitive that fixations should be generated by the same neuronal circuit controlling smooth pursuits with fixations being a special case of a target moving at zero velocity. This is probably incorrect (Leigh & Zee, 1991, pp. 139–140). Fixations, instead, are characterized by the miniature eye movements: tremor, drift, and microsaccades.

This is contrasted by, for example, work in mobile eye tracking. Jovancevic-Misic & Hayhoe [41] had participants walk around a circular path while pedestrians walked in the other direction. They state that:

Fixations were determined using manual frame-by-frame coding from the 30 Hz video record. To allow for the noise in the eye tracking signal, fixations were scored as being on the pedestrian if they fell within a 0.75° window on either side of the pedestrian. Because the pedestrians typically displaced laterally with respect to the subject during the period gaze was maintained, we treated a ‘fixation’ as the period when gaze remained at the same location with respect to the pedestrian, although it included some smooth rotational component and is more properly specified as ‘gaze’.

From a data-analysis perspective, the approach by Jovancevic-Misic & Hayhoe [41] makes sense (and it is clearly motivated at that). If the interest is in whether the pedestrians are looked at, it does not matter whether the pedestrians or the participant moves (or both). Moreover, separating pedestrian from participant movement complicates data analysis for an already time-consuming process. Indeed this reasoning seems to be more common in mobile eye-tracking studies. Steil *et al.* [56], for example, state that ‘In this work we use the term fixation to jointly refer to users’ visual focus of attention … on a gaze target irrespective of scene and head motion' (p. 2). In later work by one of the authors of the Jovancevic-Misic & Hayhoe [41] study, episodes of smooth pursuit were separated from fixations because both were the topic of research [57].

The definition of fixation by Leigh & Zee [11] has undoubtedly served the fields of, for example, neurophysiology and eye-movement dynamics very well, where separating fixation of a stationary object from moving objects is grounded in the underlying neurobiology. This definition, however, seems to be counterproductive in the new world of mobile eye tracking, including eye tracking in augmented or virtual realities. If one wants a definition of fixation that is useful across the entire breadth of current-day eye-tracking research, it is necessary to have a robust one. Or, if one all-encompassing definition is impossible, a transparent way of defining fixations and saccades. A way that allows researchers with tower-mounted eye trackers to discuss eye movements with researchers who have their participants perform extreme sports while wearing a mobile eye tracker. In our opinion, it is not worthwhile clinging to certain definitions, simply because they have been used for a long time. Nor are we interested in deciding which definition is ‘right’. In this article, we are merely interested in providing the tools for researchers to be explicit about what they consider to be a fixation, a saccade or any other event. As Gibson [58] states: ‘A good theory justifies a great deal of linguistic inconvenience; and in the long run a good theory will prevent more accidents than a muddled theory’ (p. 131).

### 4.2. Defining fixations and saccades: advice

We identify four components here that we believe are useful for qualifying fixations, saccades or other eye movements. We encourage authors of future eye-tracking studies to specify these components when defining fixations or saccades.

(1) The eye movement may be fast (e.g. saccadic movements) or slow (e.g. smooth pursuit movements). This may be considered as the oculomotor component. Note that fast and slow are relative. If one is interested in distinguishing smooth pursuit movements from the fixation of a stationary object relative to the world of a stationary observer, the former *may* be fast compared to the latter. Exactly what is considered fast or slow may be specified by giving the exact classification functions.
(2) The eye movement may serve to maintain the present area of the visual scene or object on the fovea (e.g. fixation of a stationary object, smooth pursuit or possibly even saccadic pursuit), or serve to bring a new area of the visual scene or object to the fovea (e.g. saccades to a new area of the visual scene). This may be considered as the functional component. Note that there are other functional descriptions as well, e.g. that fixations make the processing of visual information possible. Whenever such descriptions are relevant, we encourage researchers to specify them.
(3) As already implicitly addressed in the previous components, it should be clear in what coordinate system eye movements are specified. Is one discussing fixation of an object with respect to the world (world-centred coordinates), or with respect to the observer (head-centred coordinates)?
(4) It should be specified how the eye movement in question is represented in the eye-tracker signal, i.e. what computational definition is used to classify the eye movement in the eye-tracker signal? If other signals such as those from a GPS or gyroscopic system are used, specify this as well.

It is important to emphasize that definitions of fixations and saccades are likely to be context-dependent. Specifying the oculomotor event, the function, the coordinate system and a computational definition may not always be possible, for example, when the function is the topic of investigation. However, we encourage researchers to clarify and specify wherever possible. Precise definitions of fixations and saccades at the conceptual level are, for example, necessary in order to allow us to move forward in specifying better computational definitions (i.e. event classification of fixations and saccades). How are fixations classified in the eye-tracker signal by a machine-learning algorithm (e.g. [50]) meaningful, without a well-grounded definition of fixations?

Specifying multiple components of definitions of fixations and saccades is not a revolutionary idea in eye-movement research. There are examples in the literature where descriptions of eye movements were highly specific. For example, Collewijn *et al.* [59] presented examples of the eye-tracker signal from a then newly developed method. They refer to the eye-tracking data examples as: ‘Examples of (curvilinear) recordings of horizontal and vertical eye movements during fixation of an oscilloscope spot.’ Here, the main behaviour of interest is the fixation of a spot that can either be stationary or moving with respect to the observer. This leads to the following situations: ‘Fixation of immobile spot … Smooth (and additional saccadic) pursuit of circular movement of the spot … Saccadic pursuit of stepwise displacements of spot …’ (p. 449). Other good examples are by Steinman *et al.* [60], who, for example, state: ‘fixation of a target stationary with respect to his head while both head and target oscillated horizontally with respect to the environment’ (p. 103) or Collewijn & Tamminga [61] who consistently refer to the ‘fixation of a stationary target’ (p. 218). Note that one may assert that in these examples, ‘fixation’ is the instruction to the participant, rather than some eye-movement ‘event’. Our comment would be that this only underlines the need to be unambiguous in which definition is employed.

In order to give some guidance to researchers in applying this procedure, we revisit the scenarios from the introduction and provide fixation definitions for them.

#### 4.2.1. Scenario 1: eye movements during reading

Researcher A investigates gaze behaviour of adults during reading and uses an EyeLink 1000 plus eye tracker for this. The visual stimulus (the text) is static in world-centred coordinates. The gaze position reported by the eye tracker is also in world-centred coordinates. This means that the point of regard can be expressed as being on a specific part of the text. As the researcher uses the EyeLink 1000 in tower mode, the head was restrained using a chin and forehead rest. Therefore, a relatively still gaze position corresponds to a relatively still orientation of the eyes relative to the head. In this case, the following fixation definition will suffice:

A fixation is a period of time during which a specific part of the text on the screen is looked at and thereby projected to a relatively constant location on the retina. This is operationalized as a relatively still gaze position in the eye-tracker signal implemented using the EyeLink algorithm (host software v. 5.09) with the Psychophysical configuration as outlined in the EyeLink 1000 plus manual 1.0.12.

#### 4.2.2. Scenario 2: eye movements of infants

Researcher B investigates gaze behaviour of infants looking at pictures and videos of toys and uses a Tobii TX300 remote eye tracker for this. Researcher B wants to know which toy is looked at when multiple static toys are presented, and when infants track a moving toy. Thus, the object of interest (toys) can be both static and moving in world-centred coordinates. When the object is moving, the trajectory is known in world-centred coordinates. The gaze position reported by the eye tracker is also in world-centred coordinates. This means that the point of regard can be expressed as being on a specific static or moving toy. Fixations of objects includes fixation and pursuit in this scenario. The infant is placed in a baby seat, which does not fully restrain the movement of the infants' head. Therefore, a relatively still gaze position does not need to correspond to a relatively still orientation of the eyes relative to the head. In this case, the following fixation definition is used:

A fixation is a period of time during which a static (i.e. non-displacing) part of the visual stimulus (the toys) on the screen is looked at and thereby projected to a relatively constant location on the retina. This is operationalized as a relatively still gaze position in the eye-tracker signal implemented using the algorithm by Larsson *et al.* [40] with default settings. This notably excludes periods of smooth pursuit, during which a moving part of the visual stimulus on the screen is looked at and thereby projected to a relatively constant location on the retina. This is operationalized as a steadily changing gaze position in the eye-tracker signal implemented using the algorithm by Larsson *et al.* [40] with default settings, implemented by the authors and available from [website].

#### 4.2.3. Scenario 3: eye movements during ambulatory vision

Researcher C investigates gaze behaviour of a teacher in a classroom and uses an SMI ETG 2 60 Hz mobile (i.e. head-mounted) eye tracker for this. The mobile eye tracker reports point of regard in head-centred coordinates. The visual stimulus (e.g. the classroom) is, however, not described in the head-centred coordinates. Moreover, in this scenario, there is no coordinate transformation method from head-centred to world-centred frames available. This can be problematic for data analysis as the researcher wants to know the point of regard in world-centred coordinates: is the teacher looking at a student or at his desk? A useful operationalization here is that all movements of the eyes that are relatively slow (as compared to a saccadic eye movement) correspond to periods of gaze at objects. Such a slow eye movement may occur, for example, when the teacher's head is fixed while he is looking at a moving student or when the teacher moves his head while fixating his desk. When both the teacher and object looked at do not move, there is almost no movement of the eyes relative to the head. Researcher C uses a method to classify periods of slow movement of the eyes based on the eye-tracker signal, which are aptly termed ‘slow phases’. Researcher C then manually determines where the eyes of the teacher are aimed at during each slow phase. In almost all cases, Researcher C identifies an object looked at; e.g. a student walking in the classroom, or homework on a student's desk. The transformation of the point of regard from head-centred to world-centred coordinates is skipped, and resolved pragmatically by manually mapping all slow phases to the visual stimulus. To accomplish this, the researcher uses software in which frames of the scene video are presented with the average location of the slow phase superimposed. The researcher then assigns a pre-defined category (e.g. a student or desk) to the slow phase. The definition of a fixation applied here:

A fixation is a period of time during which an area of the visual stimulus is looked at and thereby projected to a relatively constant location on the retina. This is operationalized as all slow phases in the eye-tracker signal classified using the algorithm described by Hooge & Camps [62] with default settings.

The scenarios we have sketched above may seem quite extensive. However, while the context that researchers work in may seem obvious to the authors themselves, it will likely be unknown to part of the readers. Moreover, the context in which a study was conducted may be lost in a few years. We would therefore rather encourage researchers to overspecify than to underspecify their definitions. Before we conclude, we would like to emphasize again that although we focused extensively on the concepts of saccades, and particularly fixations, many of the issues we address likely apply to concepts such as *dwell*, *glance*, *gaze* and *transition* as well. Let us consider a dwell, which is defined by Holmqvist *et al.* [9] as: ‘one visit in an AOI [area of interest], from entry to exit’ (p. 190). A dwell may, for example, contain a number of fixations (if the dwell is fixation-based and not sample-based), and the problems with defining fixations therefore apply to the dwell too. The same holds for the *transition*, defined by Holmqvist *et al.* [9] as: ‘the movement from one AOI to another’ (pp. 190–191). We hope that future studies will take into consideration the potential sources of confusion highlighted by us (frames of reference, different levels of definition and event classification) when working with any eye-tracking measure. Moreover, we urge researchers working with eye movements in all research fields to be critical of ‘fixations’, ‘saccades’ or other eye-tracking measures when presented as the output of an algorithm of software without considering what these terms actually refer to and we hope that researchers will provide definitions in their papers that aim to avoid confusion through careful specification.

## 5. Conclusion

From the literature, it seems that the eye-movement field is suffering from confusion on the concepts of fixations and saccades. This was confirmed in a survey among 124 eye-movement researchers. We advise all future studies in the eye-movement literature to be more explicit when defining fixations and saccades. These definitions are context-dependent and one cannot therefore assume that definitions of such ‘well-known concepts’ as fixations and saccades are unnecessary. We urge researchers to make their definitions more explicit by specifying all known components of the definition of the eye movement under investigation, e.g. (1) the oculomotor component: e.g. whether the eye moves slow or fast; (2) the functional component: what purposes does the (lack of) eye movement serve; (3) the coordinate system used: relative to what does the eye move; and (4) the computational definition: how is the event represented in the eye-tracker signal. Although not all of these components may be known, for instance, because they are the topic of the investigation, specifying what *is* known should reduce the probability of causing conceptual confusion. Moreover, this should allow eye-movement researchers from different fields to read and discuss each other's work without ending up in misunderstanding about the eye-movement concepts discussed.

## Supplementary Material

Survey data

## Footnotes

1 Although the words themselves may remain unchanged, the meaning of concepts may have changed.

2 The Eyelink 1000 tower-mounted system is common in reading research as well. The same general scenario holds.

3 Based on Google Scholar records of January 2018, Rayner [4] has been cited over 5900 times, Leigh & Zee [11] have been cited over 4100 times, Duchowski [35] has been cited over 3000 times, Salvucci & Goldberg [32] and Holmqvist *et al.* [9] over 1100 times, and 7 of the remaining 12 on average a couple of hundred times.

4 Note that this does not necessarily mean that researchers thought PSOs should be coded as a saccade either.

## Data accessibility

The data supporting this paper have been uploaded as part of the electronic supplementary material.
